# Two-Hit *in vitro* T-Cell Stimulation Detects *Mycobacterium tuberculosis* Infection in QuantiFERON Negative Tuberculosis Patients and Healthy Contacts From Ghana

**DOI:** 10.3389/fimmu.2019.01518

**Published:** 2019-07-03

**Authors:** Ernest Adankwah, Christian Lundtoft, Alptekin Güler, Kees L. M. C. Franken, Tom H. M. Ottenhoff, Ertan Mayatepek, Ellis Owusu-Dabo, Richard Odame Phillips, Norman Nausch, Marc Jacobsen

**Affiliations:** ^1^Department of General Pediatrics, Neonatology, and Pediatric Cardiology, University Children's Hospital, Medical Faculty Heinrich-Heine University, Düsseldorf, Germany; ^2^Department of Immunohematology and Blood Transfusion, Department of Infectious Diseases, Leiden University, Leiden, Netherlands; ^3^Kumasi Centre for Collaborative Research in Tropical Medicine, Kumasi, Ghana; ^4^School of Public Health, College of Health Sciences, Kwame Nkrumah University of Science and Technology, Kumasi, Ghana

**Keywords:** tuberculosis, LTBI, IGRA, *Mycobacterium tuberculosis* latency antigens, ESAT6, CFP10

## Abstract

IFN-γ release assays [e.g., QuantiFERON (QFT)] are widely used for diagnosis of *Mycobacterium tuberculosis* (*Mtb*) infection. T-cell responses against QFT antigens ESAT6 and CFP10 are highly *Mtb* specific but previous studies indicated suboptimal assay sensitivity. Especially for potentially infected healthy contacts (HCs) of tuberculosis patients, alternative antigen usage and more sensitive tests may contribute to improved detection of latent *Mtb* infection. In a pilot case-control study of tuberculosis patients (*n* = 22) and HCs (*n* = 20) from Ghana, we performed multifaceted *in vitro* assays to identify optimal assay conditions. This included a two-hit stimulation assay, which is based on initial and second re-stimulation with the same antigen on d6 and intracellular IFN-γ analysis, to compare T-cell responses against ESAT6/CFP10 (E6/C10) and selected latency antigens (i.e. Rv2628, Rv1733, Rv2031, Rv3407) of *Mtb*. Considerable subgroups of tuberculosis patients (64%) and HCs (75%) had negative or indeterminate QFT results partially accompanied by moderate PHA induced responses and high IFN-γ background values. Intracellular IFN-γ analysis of E6/C10 specific CD4^+^ T-cell subpopulations and evaluation of responder frequencies had only moderate effects on assay sensitivity. However, two-hit *in vitro* stimulation significantly enhanced E6/C10 specific IFN-γ positive T-cell proportions especially in QFT non-responders, and in both study groups. *Mtb* latency antigen-specific T cells against Rv1733 and Rv2628 were especially detected in HCs after two-hit stimulation and T-cell responses against Rv2628 were highly capable to discriminate tuberculosis patients and HCs. Two-hit *in vitro* stimulation may improve moderate sensitivity of short term IFN-γ based assays, like QFT, to detect *Mtb* infection. Latency stage-specific antigens added significantly to detection of *Mtb* infection in HCs and tuberculosis patients with negative QFT test results.

## Introduction

Tuberculosis is a chronic infectious disease caused by *Mycobacterium tuberculosis* (*Mtb*). The pathogen is transmitted via aerosols from tuberculosis patients with cavernous disease to close contacts (HCs), who have a high risk of becoming *Mtb* infected. A minor subset of *Mtb* infected HCs will develop active tuberculosis whereas the majority will control the pathogen by immune surveillance and remain latently *Mtb* infected (LTBI) (1). IFN-γ producing CD4^+^T_helper_ cells are central for protection of LTBI against progression to active disease. T_helper_ cells induce a delayed type hypersensitivity reaction against mycobacterial antigens (i.e., *Mtb* purified protein derivative; PPD) and this recall immune response forms the basis of the tuberculin skin test (TST) for detection of previous *Mtb* infection. The TST test has been used for more than a century to diagnose *Mtb* infection. In several regions where tuberculosis is endemic the TST is still applied but replacement by more specific immunological *in vitro* tests (i.e., IFN-γ release assays, IGRAs) is ongoing (2).

Immunological tests are essential for diagnosis of *Mtb* infection since direct detection of *Mtb* in affected body fluids is only possible for a subgroup of tuberculosis patients and generally not for LTBI. Identification of LTBI within HCs, however, is crucial especially for individuals with high risk of tuberculosis disease progression (e.g., young children) (3). IGRAs, like the QuantiFERON® Gold Plus test (QFT) are based on IFN-γ measurement after *in vitro* stimulation of whole blood with selected *Mtb* antigens (i.e., Early Secretory Antigenic Target (ESAT)-6 and Culture Filtrate Protein (CFP)-10). IGRAs can be assessed faster (i.e., after 16h) with TST comparable sensitivity but higher specificity especially in BCG vaccinated individuals (4, 5). Own previous studies showed that QFT sensitivity varies markedly between children with tuberculosis from different regions, being high in children with tuberculosis from Germany and alarmingly low in children from Ghana (6, 7). The usage of few selected antigens may contribute to population-dependent differences in QFT sensitivity due to differential MHC or exposure background. In addition, QFT antigens are predominantly expressed during active stage of *Mtb* and may only partially reflect host immune response against *Mtb* at different stages (i.e., dormancy, reactivation, resuscitation) (8). The term “latency antigens” of *Mtb* has been widely used for non-active stage antigens and is adopted here.

Several studies investigated T-cell response against *Mtb* latency in study groups of tuberculosis patients and controls [reviewed in (9)]. These studies were either using whole blood (10–13) or purified peripheral blood mononuclear cells (PBMC) (10, 14–16) in short-term (i.e., 16–24 h) (11, 13–15) or long-term (i.e., 5–7d) (11, 12, 14, 16, 17) assays measuring IFN-γ in the supernatant (10–17) or intracellularly (10, 11, 13). Only few studies compared different assays and found marked differences of individual donors between short- and long-term assays (18–21). We previously demonstrated that long-term culture (i.e., 7 days) including a second stimulation with the respective antigen on d6 (two-hit) and intracellular IFN-γ analysis enhanced the sensitivity for detection of T-cell responses against latency antigens (19). To investigate the immunogenicity of latency antigens, the vast majority of studies were based on QFT positive individuals, especially for LTBI. However, this excludes significant subgroups of both tuberculosis patients and LTBI, that may well show false-negative QFT results. Especially for HCs, early identification of *Mtb* infection is crucial to avoid spread of tuberculosis in endemic countries like Ghana.

Latency antigen specific T-cell responses may especially be important as a biomarker for LTBI, where *Mtb* dormancy contributes to pathogen survival (22). These T cells are, therefore, not only potential indicators of *Mtb* infection but may contribute to immune surveillance crucial to avoid *Mtb* reactivation and disease progression.

This pilot study aims at improving *in vitro* culture conditions to detect T-cell responses against QFT antigens ESAT6 and CFP10 (E6/C10) and selected latency antigens (i.e., Rv2628, Rv1733, Rv2031, Rv3407) in well-characterized tuberculosis patients and HC cohorts. A special focus was on tuberculosis patients with negative (or indeterminate) QFT results. For these we evaluated the capacity of alternative assays and antigens for detection of *Mtb* infection. Furthermore, the efficacy of latency antigen-specific T-cell based tests to classify tuberculosis patients and HCs was investigated.

## Materials and Methods

### Study Cohort

We recruited tuberculosis patients (*n* = 22) and HCs (*n* = 20) from August to October 2018 at the Presbyterian Hospital in Agogo/Ghana. An independent cohort used for classification analyses [i.e., tuberculosis patients (*n* = 4) and HCs (*n* = 13)] was recruited between January and February 2016 at the Komfo Anokye Teaching Hospital, Kumasi/Ghana.

Diagnosis for active tuberculosis was based on patient history, clinical evaluation, chest X-ray, and sputum smear test. GeneXpert (Cepheid, USA) analyses were done for the main tuberculosis study group. 15 out 22 tuberculosis patients were GeneXpert-positive. GeneXpert negative tuberculosis patients had chest X-ray and clinical symptoms (i.e., blood coughing, weight loss) that were strongly suggestive for tuberculosis. 4 of 7 GeneXpert negative tuberculosis patients had positive QFT results. Sputum culture is not routinely performed. All tuberculosis patients of the second “classification” cohort were sputum smear positive for Mtb and no GeneXpert was performed for those. All tuberculosis patients were included prior to initiation of treatment and chemotherapy has been initiated immediately thereafter. The vast majority of tuberculosis patients had pulmonary disease manifestation besides two children, who had lymph node tuberculosis. HIV infection was excluded for all participants.

HCs had no symptoms of tuberculosis but were close relatives living in the same household with indexed tuberculosis patients according to self-report and direct observation. Study group characteristics are shown in Table 1.

**Table 1:** Study cohort characteristics.

	**Group**	***n***	**Median age (range, y)**	**Gender (m/f)**	**Tested conducted**		
					**QFT**	**X-ray**	**Sputum smear (microscopy or GeneXpert)**
Study cohort	TB	22	37 (3–74)	18/4	√	√	√ (15 GeneXpert positive^*^)
	HC	20	33.5 (5–61)	12/8	√	nd	nd
Test cohort	TB	4	61(13–73)	3/1	√	√	√ (all microscopy positive)
	HC	13	36 (20–76)	8/5	√	nd	nd

Y, years; m, male; f, female, n, numbers, nd, not done; TB, tuberculosis patients; HCs, healthy controls;

* *Tests included GeneXpert analyses; four of seven GeneXpert negative TB patients were QFT positive*.

### Ethical Statement

The present study received approval from the Committee on Human Research, Publication and Ethics (CHRPE/AP/023/18; CHRPE/221/14) at the School of Medical Sciences (SMS) at the Kwame Nkrumah University of Science and Technology (KNUST) in Kumasi, Ghana. All study subjects gave written informed consent prior to recruitment and for children written informed consent was provided by their parents or legal guardians.

### QFT and QFT_*in-vitro*_ Assays

Whole blood was taken in a single venepuncture into heparin tubes and 0.8 ml were added to each of the four QFT tubes (i.e., Nil, TB1, TB2, and PHA; Qiagen). The tubes were immediately incubated at 37°C for about 20 h. For the QFT_*in-vitro*_ assay, 100 μl whole blood was cultured in 100 μl RPMI supplemented with, Penicillin/Streptomycin (100 U/ml) and L-glutamine (2 mM) using a 96 U-bottom plate (Greiner). Samples were stimulated with recombinant ESAT6/CFP10 fusion protein (E6/C10: 2 μg/ml), purified protein derivative of *Mtb* (PPD: 10 μg/ml; Statens Serum Institute), phytohemagglutinin (PHA: 10 μg/ml; Sigma-Aldrich) or left unstimulated for 20 h at 37°C and 5% CO_2_. The term QFT_*in-vitro*_ has been introduced since the results serve as reference between QFT and ICS-based *in vitro* assays (see below). In contrast to QFT, the QFT_*in-vitro*_ uses recombinant proteins (not optimized peptide mixtures) and blood dilution. However, for comparison of PPD stimulation, as well as PBMC and intracellular cytokine staining (ICS), this QFT comparable assay was needed.

Supernatants from both assays were harvested and stored at −80°C until further analysis.

### Measurement of IFN-γ Concentrations in the Supernatant

Supernatants from QFT and QFT_*in-vitro*_ assay were thawed and IFN-γ concentrations were measured by ELISA (R&D) according to manufacturer's instruction. All samples were analyzed in duplicate and measured using an Infinite M200 ELISA reader (Tecan). Concentrations were calculated from respective standard curves by applying 4-parametric logistic regression. IFN-γ concentration of non-stimulated controls were subtracted from *Mtb* antigen specific and PHA induced IFN-γ to retrieve Δ values. Values below 1 pg/ml were set to 1 pg/ml. A previously described algorithm for the interpretation of IFN-γ values based on manufacturer's criteria was used for comparison of QFT (Table S1). These criteria have been adjusted for evaluation of QFT_*in-vitro*_ results (Table S2) (6). QFT includes two tubes (TB1, TB2 according to manufacturer's nomenclature) containing *Mtb* antigens optimized for CD4^+^ and CD8^+^ T-cell response, respectively. Since both tubes showed similar results (data not shown) only TB1 (termed Ag1 in this manuscript) has been included for comparisons.

**Figure 1:**
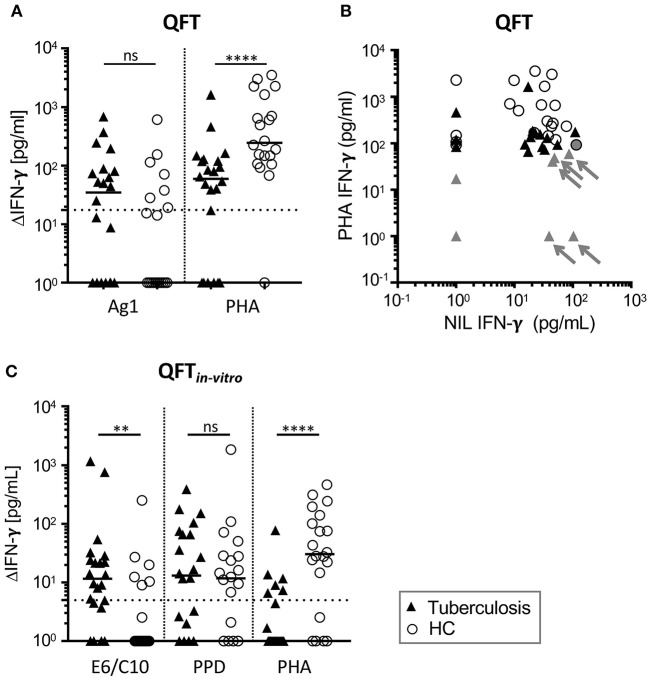
High proportions of QFT and QFT_*in-vitro*_ negative (or indeterminate) results from tuberculosis patients and healthy contacts (HCs). IFN-γ concentrations in supernatants of QFT **(A,B)** or the QFT_*in-vitro*_ **(C)** whole blood overnight culture shown for tuberculosis patients (TB) (*n* = 22) and HCs (*n* = 20). Symbols indicate mean values of duplicates for individual tuberculosis patients (black triangles) and HCs (open circles). **(A,C)** Results for antigen-specific stimuli [Ag1 for QFT; ESAT6/CFP10 recombinant fusion protein (E6/C10), purified protein derivative of *Mtb* (PPD)] and PHA were subtracted for IFN-γ measured in the unstimulated sample (ΔIFN-γ). Significant differences are indicated by asterisks. Nominal *p*-values for the Mann-Whitney *U*-test (two-tailed) were calculated and shown as **p < 0.01 and ****p < 0.0001. ns: not significant. **(B)** NIL (x-axis) and PHA (y-axis) induced IFN-γ concentrations of QFT supernatants are shown for TB patients and HCs. Individuals with indeterminate QFT results are indicated by gray color. Arrows indicate donors evaluated as indeterminate because of high IFN-γ NIL values and weak PHA response. PHA, phytohemaglutinin; NIL, Non-stimulated sample.

Indeterminate test results from QFT assay can have different explanations (e.g., NIL>400 pg, see Table S1) and the underlying cause for indeterminate tests cannot be deduced from single parameters. For this reason, we did not include evaluation results as different symbol colors in Figure 1A but depict effects of high NIL values in combination with PHA response on indeterminate QFT results in Figure 1B.

### Short-Term Intracellular IFN-γ Assays: ICS_Blood_ and ICS_PBMC_

Peripheral blood and PBMCs were used concomitantly. PBMCs were isolated from heparinized whole blood according manufacturer's instruction using density centrifugation (Ficoll, Biochrom). PBMCs (1.2 × 10^5^) were cultured in RPMI (200 μl) and heparinized whole blood (100 μl) was diluted 1:1 in RPMI. Co-stimulatory antibodies were applied to optimize detection of *Mtb* specific T cells in this assay (23). All wells received co-stimulatory antibodies αhuman CD49d (clone 9F10) (1 μg/ml) and αhuman CD28 (clone CD28.2) (1 μg/ml). PPD (10 μg/ml), E6/C10 (2 μg/ml), or PHA (10 μg/ml) were added to the indicated samples. PBMC samples were also stimulated with *Mtb* latency antigens Rv2628 and Rv3407 (each 2 μg/ml) and were generally supplemented with human recombinant IL-7 (5 ng/ml). Samples were incubated for 2 h prior to addition of Brefeldin A (2.5 μg/ml; Sigma Aldrich). Subsequently, samples were incubated for about 20 h at 37°C and 5% CO_2_. Afterwards, erythrocytes were lysed (RBC lysis buffer; Sigma Aldrich) for the whole blood assay. Samples were then fixed, permeabilized and stained with the following panel of αhuman antibodies: CD3 APC (clone UCTH1), CD4 PerCP/Cy5.5 (clone RPA-T4), IFN-γ PE (clone B27) and TNF-α FITC (clone Mab 11) (all Biolegend). Cells were acquired using BDAccuri C6 flow cytometer (BD Biosciences). The median number of CD4^+^ T cells analyzed was 6142 (range: 1,120–20,671) for tuberculosis patients and 11,097 (range: 3,375–48,661) for HCs. The data were analyzed by FlowJo software (Version 10, FlowJo LLC). A representative example for the gating procedure is depicted in Figure S1. A threshold of 0.03 % IFN-γ producing CD4^+^ T cells (three times higher than the assumed detection limit of 0.01% for flow cytometry) was set for classifying an individual donor as a “responder.”

### Two-Hit Assay

The two-hit assay has been performed [as described previously (19)] with minor modifications. In brief, 10^5^ PBMCs were cultured in 200 μl RPMI supplemented with Penicillin/Streptomycin (100 U/ml), L-glutamine (2 mM), 10 mM HEPES buffer (all Gibco), 7.5% human serum (Sigma Aldrich) and 5 ng/ml of recombinant IL-7 (Sigma-Aldrich) in 96-U bottom plate. Cells were stimulated or left unstimulated with two *Mtb* antigens PPD (1μg/ml), ESAT6/CFP10 fusion protein (E6/C10; 1 μg/ml) and four latency antigens (i.e., Rv1733, Rv2628, Rv2031, and Rv3407; 1μg/ml). E6/C10 as well as the *Mtb* latency antigens are recombinant proteins produced in the laboratory of K. Franken and have been thoroughly used in previous studies (18, 19, 24). Samples were then incubated for 6 days at 37°C and 5% CO_2_. On the sixth day, 100 μl of culture supernatants were discarded and samples were re-stimulated with the respective *Mtb* antigens (same concentrations as on d1) and Brefeldin A (3.75 μg/ml) (Sigma Aldrich) in reconstituted in fresh medium for 20 h. Afterwards, cells were then fixed, permeabilized and stained with the following panel of antibodies: CD3 APC (clone UCTH1), CD4 PerCP/Cy5.5 (clone RPA-T4), IFNγ PE (clone B27) and TNF-α FITC (clone Mab 11) (all BioLegend). Cells were measured as described above.

### Statistical Analyses

Statistical analysis was performed using GraphPad prism Software v7 (Graphpad Software). A non-parametric Mann Witney U and Wilcoxon matched-pairs rank test were applied and indicated in the according figure legends. The Spearman Rank test was used to determine significant correlations between E6/C10 specific CD4^+^ T-cell proportions and participant age for all donors and both study groups separately. Correlation coefficients (r) and nominal p-values are given. Receiver Operating Characteristic (ROC) curve analysis was performed to evaluate the diagnostic performance of *Mtb* and latency antigens in the discrimination of active tuberculosis from HCs. Ternary plots were generated to determine qualitative difference of T-cell responses against *Mtb* E6/C10 and latency antigens using Grapher Software (Golden Software, LLC). Significance was considered at a *p*-value of ≤ 0.05.

## Results

### Low Sensitivity of the QFT in Tuberculosis Patients and HCs

We performed QFT of tuberculosis patients (*n* = 22) and healthy contacts (*n* = 20) from Agogo in Ghana. Eight patients with tuberculosis (36%) and five contacts (25%) had positive QFT results (Table 2) according to manufacturer's criteria (Table S1). Notably, the majority of tuberculosis patients and contacts were negative or indeterminate (Table 2). Indeterminate results were more frequent in tuberculosis patients (32%) as compared to healthy contacts (5%) (Table 2). Generally impaired T-cell response of tuberculosis patients has been described and we found lower PHA-induced IFN-γ of tuberculosis patients (Figure 1A). In addition, differences in IFN-γ background (NIL) levels may affect result interpretation since NIL-subtracted Δ values are used for QFT evaluation (Table S1). The majority of donors had detectable IFN-γ values in the NIL sample and, in combination with impaired T-cell response of tuberculosis patients to PHA (Figures 1A,B), NIL IFN-γ_high_ background caused indeterminate QFT results in a subgroup of tuberculosis patients (Figure 1B). To investigate possible effects of NIL IFN-γ_high_ background on positive QFT results, we compared proportions of QFT positive individuals between tuberculosis patient and HC subgroups (i.e., NIL IFN-γ_high_ vs. NIL IFN-γ_low_). Comparable proportions of NIL IFN-γ_high_ individuals between QFT positive and negative/indeterminate individuals did not indicate NIL IFN-γ effects on positive test results (Figure S2). We concluded that QFT diagnosed *Mtb* infection only for a minority of tuberculosis patients and HCs. In addition, impaired T-cell response in tuberculosis patients in combination with high NIL_IFN_-γ levels hampered interpretation of QFT results.

**Table 2:** QFT and QFT_*in-vitro*_ evaluation of test results.

	**QFT**			**QFT**_***in***−***vitro***_		
	**Positive *n* (%)**	**Indeterminate *n* (%)**	**Negative *n* (%)**	**Positive *n* (%)**	**Indeterminate *n* (%)**	**Negative *n* (%)**
TB (*n* = 22)	8 (36%)	7 (32%)	7 (32%)	9 (41%)	9 (41%)	4 (18%)
HC (*n* = 20)	5 (25%)	1 (5%)	14 (70%)	4 (20%)	4 (20%)	12 (60%)

*n, number*.

### A QFT_*in-vitro*_ Assay Confirmed Marginal Sensitivity of QFT to Detect *Mtb* Infection

Since GeneXpert analyses confirmed *Mtb* infection for the majority of tuberculosis cases (including six of seven patients with indeterminate QFT results), we concluded that QFT results were at least partially false negative. Suboptimal assay conditions may lead to low assay sensitivity as a possible reason for negative test results. To analyse this, we established an *in vitro* assay based on recombinant *Mtb* antigen-specific *in vitro* culture and IFN-γ measure. Initially, we applied QFT comparable conditions and measured IFN-γ concentrations in the supernatant of E6/C10, PPD, and PHA stimulated T cells (QFT_*in-vitro*_). IFN-γ concentrations were generally lower in the QFT_*in-vitro*_ assay as compared to the QFT (Figure 1) and E6/C10 specific IFN-γ concentrations in HCs were significantly lower as compared to tuberculosis patients (Figure 1C; *p* = 0.004). To facilitate direct comparison of both assays, we adjusted the threshold of positive results for the QFT_*in-vitro*_ assay (according to PHA induced median IFN-γ; Figure 1C) and classified tuberculosis patients and contacts as positive, indeterminate, or negative in both assays (Table S2). Proportions were largely comparable between the assays and, like for the QFT, high numbers of tuberculosis patients and HCs were indeterminate or negative in the QFT_*in-vitro*_ assay (Table 2).

### Moderately Increased PPD-Specific CD4^+^ T-Cell Proportions Detected by ICS_***in-vitro***_

**Figure 2:**
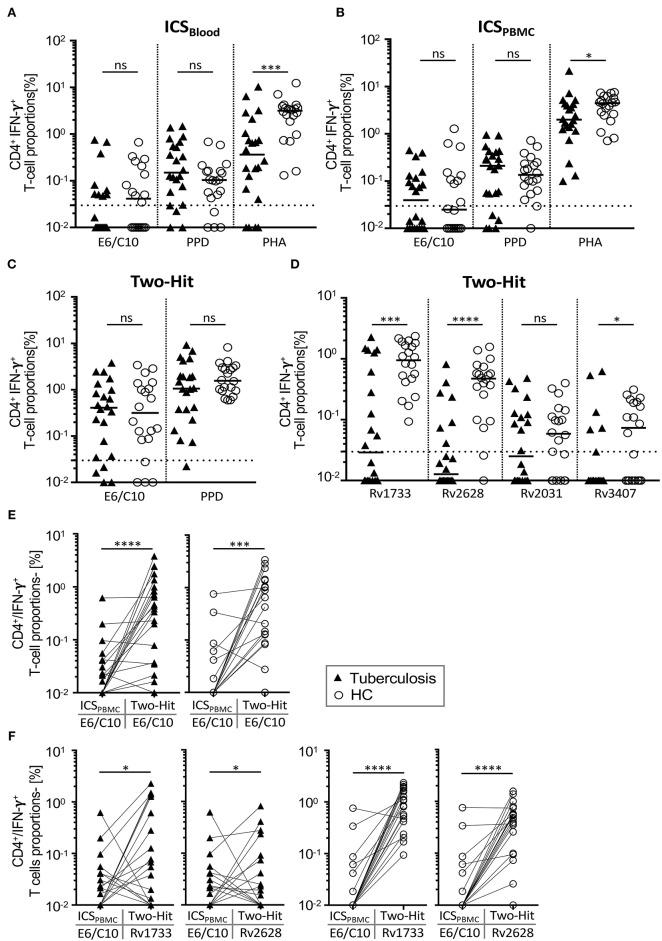
IFN-γ positive CD4^+^ T-cell proportions specific for E6/C10 and latency Mtb antigens after short-term and two-hit *in vitro* culture. IFN-γ positive CD4^+^ T cells of TB patients and HCs were measured by intracellular flow cytometry after stimulation short-term **(A,B)**, long-term culture (two-hit) **(C,D)** or comparison between both **(E,F)**. E6/C10, PPD, or PHA were used in short-term whole blood **(A)** or purified PBMCs culture **(B)**. E6/C10, PPD, and latency antigens (i.e., E6/C10, Rv1733, Rv2628, Rv2031, or Rv3407) were used in long-term two-hit stimulation **C,D**). Symbols indicate mean values of duplicates for individual tuberculosis patients (black triangles) and HCs (open circles). **(E,F)** Paired comparison of short-termE6/C10 specific CD4^+^ T-cell proportions with long-term two-hit stimulation for E6/C10 **(E)** and Rv1733, Rv2628 **(F)** in TB patients (left graphs) and HCs (right graphs). Significant differences are indicated by asterisks. Nominal *p*-values for the Mann-Whitney *U*-test (two-tailed) were calculated and shown as *p < 0.05; ***p < 0.001 and ****p < 0.0001. ns, not significant.

To exclude possible effects of bystander IFN-γ production and/or differential IFN-γ serum levels that may affect QFT and QFT_*in-vitro*_, we next performed intracellular IFN-γ measurements in *Mtb* specific T cells in whole blood (ICS_Blood_) as well as purified PBMCs (ICS_PBMCs_). Proportions of CD4^+^ IFN-γ positive T cells largely reflected results from QFT/QFT_*in-vitro*_ showing significantly higher responses for PHA in HCs as compared to tuberculosis patients for ICS_Blood_ and ICS_PBMCs_ (Figures 2A,B). To compare the obtained results between different assays, we set a threshold of 0.03% IFN-γ positive CD4^+^ T cells to classify “responders” to respective stimuli (Figures 2A,B; Table 3). Similar proportions of E6/C10 responders were found for tuberculosis patients in different assays [QFT_*in-vitro*_: 9 (41%); ICS_Blood_: 9 (41%); ICS_PBMC_: 11 (50%)] (Table 3). For HCs, the proportions of responders were higher in both ICS assays [ICS_Blood_: 11 (55%); ICS_PBMC_: 8 (40%)] as compared to the QFT_*in-vitro*_ [4 (20%)]. PPD responders were generally more frequent than E6/C10 responders (Table 3) and ICS assays detected higher proportions of PPD responders (77 to 86%) as compared to QFT_*in-vitro*_ (45 %) (Table 3). These results indicated that additional *Mtb* antigens may improve sensitivity and that all examined short-term assays showed suboptimal sensitivity to detect *Mtb* infection using E6/C10 antigen.

**Table 3:** QFT_*in-vitro*_ comparison with intracellular cytokine-based assays.

	**E6/C10 responder**			**PPD responder**		
	**QFT_***in*−*vitro***_*n* (%)**	**ICS_**Blood**_*n* (%)**	**ICS_**PBMC**_*n* (%)**	**QFT_***in*−*vitro***_*n* (%)**	**ICS_**Blood**_*n* (%)**	**ICS_**PBMC**_*n* (%)**
TB (n=22)	9 (41%)	9 (41%)	11 (50%)	10 (45%)	19 (86%)	17 (77%)
HCs (n=20)	4 (20%)	11 (55%)	8 (40%)	9 (45%)	16 (80%)	18 (82%)

*ICS, intracellular cytokine analysis; PPD, purified protein derivatives of Mtb*.

### Two-Hit Stimulation Improves Detection of E6/C10 and Latency Antigen-Specific T Cells

Previously, we demonstrated that seven days of *in vitro* culture including two-hit stimulation enhanced sensitivity for detection of IFN-γ expressing T cells and enabled identification of T-cell response against *Mtb* latency antigens (19). Importantly, this “two-hit” assay did not prime *Mtb* specific T-cell response in the absence of previous *Mtb* infection (19). Two-hit stimulation was performed with PPD, E6/C10, and selected *Mtb* latency antigens (i.e., Rv2628, Rv1733, Rv2031, Rv3407) of tuberculosis patients and HCs. Tuberculosis patients and HCs had comparable proportions of PPD- or E6/C10- specific CD4^+^ T cells (Figure 2C). Classification of responders (>0.03% IFN-γ^+^ CD4^+^ T cells) revealed that the vast majority of tuberculosis patients and HCs responded to E6/C10 in the two-hit assay (tuberculosis patients: 18 (82%); HCs 16 (80%); Figure 2C). For PPD, all HCs and 21 (96%) of the tuberculosis patients showed a positive response in the two-hit assay (Figure 2C).

For latency antigens, two-hit stimulation with Rv2628 and Rv1733 induced IFN-γ producing T cells in the majority of individuals, whereas smaller subgroups had specific T cells for Rv2031 and Rv3407 (Figure 2D). Notably, and in contrast to E6/C10, Rv2628, Rv1733, and Rv3407 specific T cells were significantly more frequent in HCs as compared to tuberculosis patients (Rv2628: *p* < 0.0001; Rv1733: *p* = 0.0003; Rv3407: *p* = 0.039; Figure 2D). Classification of responders revealed that all HCs responded to Rv1733 and 18 (90%) of HCs to Rv2628 (Figure 2D). In contrast, only 11 (50%) and 7 (32%) of tuberculosis patients were responders to Rv1733 or Rv2628, respectively (Figure 2D).

To determine whether *Mtb* latency antigens could also be used to detect *Mtb* infection in short-term assays, we selected two candidates (i.e., Rv2628, Rv3407) to perform additional ICS_PBMC_ assays. IFN-γ^+^ CD4^+^ T cells were detectable for both latency antigens (Figure S3) and proportions were comparable to E6/C10 in the same assay (Figure 2B; Table 3). As for E6/C10, however, considerable proportions of tuberculosis patients (*n* = 11, 50%) and HCs (*n* = 10, 50%) were negative for Rv2628 specific T-cell responses and even less individuals had detectable T-cell responses against Rv3407 after short-term stimulation (Figure S3). In addition, there was no significant difference between tuberculosis patients and HCs (Figure S3). We concluded that short-term stimulation was also suboptimal for *Mtb* latency antigens Rv2628 and Rv3407 and focused on the two-hit assay for further analyses.

Since individuals from both study groups varied markedly in age at recruitment, we determined possible age-dependent effects on two-hit results. No correlation was seen between age and two-hit E6/C10 responses (Figure S4A). In addition, exclusion of children/adolescents (below 18 years) from analyses had no detectable effects on two-hit results (Figure S4B). Therefore, age-dependent effects on T-cell response in two-hit assays were not found.

### QFT_*in-vitro*_ Negative Tuberculosis Patients and LTBI Are Predominantly Promoted in the Two-Hit Assay

False negative (or indeterminate) QFT results of tuberculosis patients are a major obstacle in the diagnosis of active tuberculosis especially if direct proof of *Mtb* infection is not possible. Furthermore, negative QFT results preclude identification of LTBI within HCs. To address the question whether E6/C10 results from the two-hit assay could be used for detection of *Mtb* infection in QFT negative individuals, we compared short-term ICS_PBMC_ with two-hit results (Figure 2E) and evaluated the median fold change (Table 4) for the study cohorts. A significant increase of E6/C10 specific T-cell proportions was found in the long-term two-hit assay as compared to the short-term ICS_PBMC_ assay for both study groups (tuberculosis patients: *p* < 0.0001; HCs: *p* = 0.0003; Figure 2E). Notably, ICS_PBMC_ non-responders within both study groups showed significantly enhanced E6/C10 specific T-cell responses (tuberculosis: median fold-change 33.7; HCs: median fold-change 42.8; Table 4) whereas E6/C10 ICS_PBMC_ responders were hardly affected by two-hit stimulation (tuberculosis: median fold-change: 1.2; HCs: median fold-change 1.3) (Table 4). In accordance, 10 of 13 (77 %) of the ICS_PBMC_ negative tuberculosis patients and 12 of 16 (75 %) of the ICS_PBMC_ negative HCs were classified as responders in the two-hit assay.

**Table 4:** Two-hit results for categorized strong and weak E6/C10 responders.

	**TB**		**HCs**	
**E6/C10, d1:**	**Low**	**High**	**Low**	**High**
E6/C10, fold-change median (range)	33.7 (0.4–381.1)	1.2 (0.8–31.6)	42.8 (1.0–341.2)	1.3 (0.32–10.6)
Rv1733, fold-change median (range)	5.5 (0.5–149.5)	0.4 (0.02–14.71)	106.8 (9.4–237.4)	3.3 (0.6–19.4)
Rv2628, fold-change median (range)	1.5 (0.5–41.0)	0.33 (0.02–8.5)	46.1 (1.0–139.5)	1.5 (1.0–11.7)

*R, range; fc, fold-change*.

### Short-Term Assay Non-responders Are Detected by Two-Hit Rv2628/Rv1733 Stimulation

To evaluate the capacity of Rv2628 and Rv1733 to detect *Mtb* infection in E6/C10 non-responders, we compared ICS_PBMCs_ values with the two-hit results for Rv2628 and Rv1733 between the study groups. Tuberculosis patients showed heterogeneous responses against both latency antigens compared to E6/C10 whereas HCs had markedly higher T-cell proportions against Rv1733 and Rv2628 (Figure 2F). Notably, ICS_PBMCs_ non-responders from both study groups showed a significantly stronger T-cell response against Rv1733 and Rv2628 in the two-hit assay as compared to ICS_*in-vitro*_ responders (tuberculosis patients, Rv2628 *p* = 0.03, Rv1733 *p* = 0.02; HCs, Rv2628 *p* = 0.008, Rv1733 *p* = 0.002) (Table 4). Four tuberculosis patients and three HCs were non-responders in both, E6/C10 specific ICS_PBMCs_ and two-hit, assays. Inclusion of Rv1733 two-hit results confirmed *Mtb* infection in all E6/C10 non-responder HCs of this subgroup and two of four tuberculosis patients. Therefore, inclusion of latency antigen specific T-cell responses in the two-hit assay may increase sensitivity for detection of *Mtb* infection.

### Mtb Latency Antigens Distinguish LTBIs From Tuberculosis Patients

**Figure 3:**
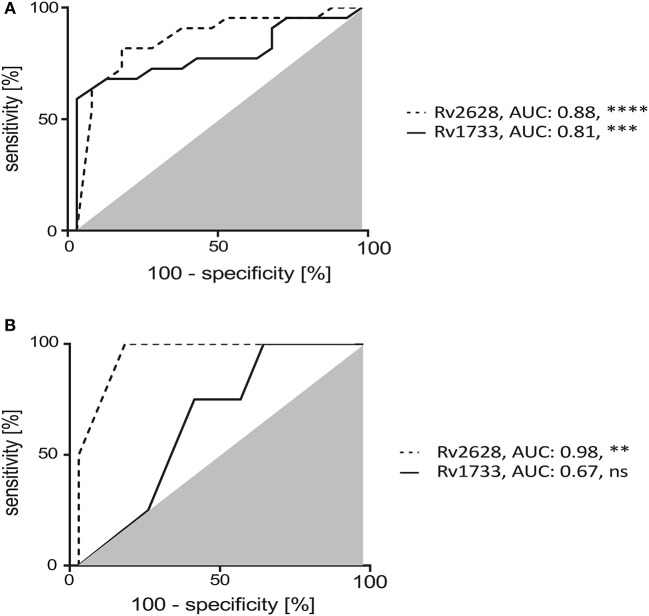
Two-hit assay induced Mtb latency antigen specific IFN-γ positive CD4^+^ T cells discriminate TB patients and HCs. Receiver Operator Characteristic (ROC) analysis for classification of TB patients and HCs using Rv1733 and Rv2628 specific IFN-γ positive CD4^+^ T cells of TB patients and HCs after two-hit stimulation. Area Under Curve (AUC) values of ROC analysis for the study groups **(A)** and an independent test cohort **(B)** [TB patients (*n* = 4) and HCs (*n* = 13)]. Deduced AUC-values and nominal *p*-values are shown as **p < 0.01, ***p < 0.001, and ****p < 0.0001. ns: not significant.

Discrimination of tuberculosis patients from HCs (especially LTBIs) has important implications e.g., for intervention strategies. Therefore, we next determined the capacities of Rv2628 and Rv1733 to classify tuberculosis patients and LTBIs. ROC analyses showed robust classification efficacy for Rv2628 (AUC: 0.88, *p* < 0.0001) and Rv1733 (AUC: 0.81, *p* = 0.0006) (Figure 3A). To validate classification results we included an independent test cohort of tuberculosis patients (*n* = 4) and HCs (*n* = 13). ROC analyses verified the discrimination capacity of Rv2628 (AUC:0.98, *p* = 0.0046) whereas Rv1733 (AUC:0.67, *p* = 0.30) was less effective in the test cohort (Figure 3B). In conclusion, T-cell responses against Rv2628 from the two-hit assay effectively discriminated HCs from tuberculosis patients.

### Mtb Antigen Specificity Pattern Differ Between QFT-Negative LTBIs and Tuberculosis Patients

**Figure 4:**
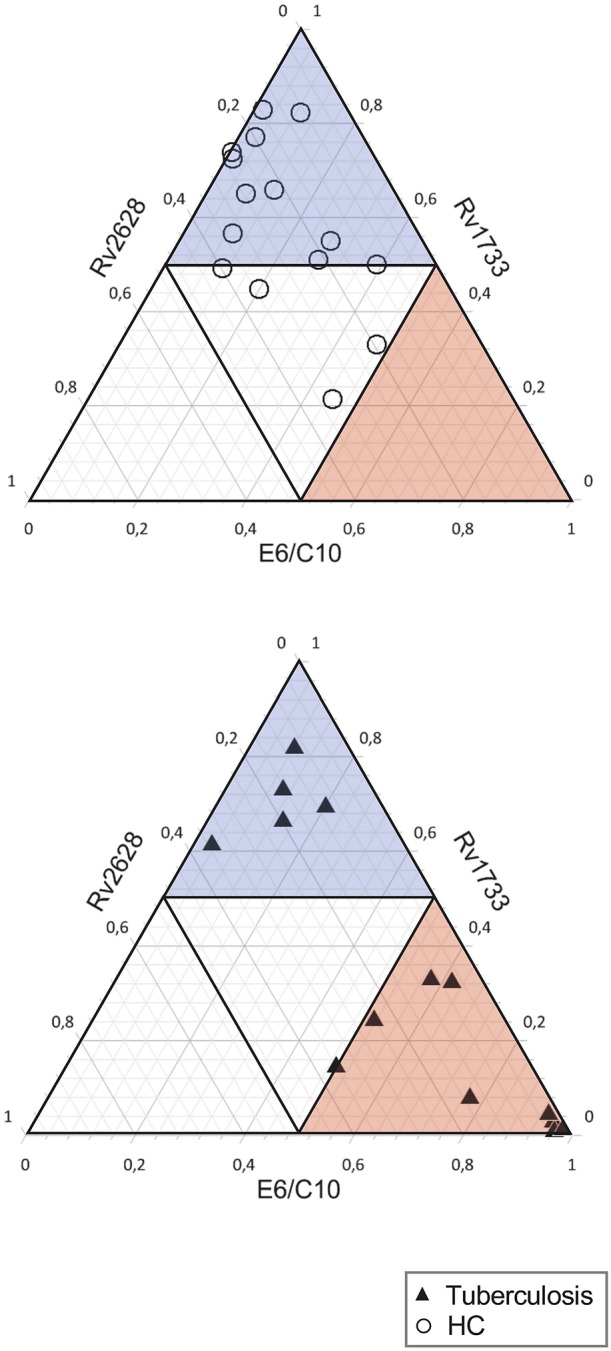
E6/C10 and Rv1733 dominant T-cell recognition pattern from the two-hit assay indicate two distinct subgroups in QFT negative (or indeterminate) tuberculosis patients. Ternary plots depict relative T-cell proportions specific for E6/C10, Rv1733, and Rv2628 of HCs (upper graph) and tuberculosis patients (lower graph) with low (indeterminate) QFT response. The sum of all IFN-γ positive T cells after two-hit stimulation with one of these antigens is set to 1. Dominant results (defined as > 50% positive T cell) are indicated as open triangle for Rv2628, blue transparent triangle for Rv1733, and red transparent triangle for E6/C10. Each symbol depicts results for an individual tuberculosis patient (black triangle) or HC (open circle).

Finally, we characterized qualitative differences of T-cell responses against E6/C10, Rv2628, and Rv1733 for QFT low (or indeterminate) individuals from both study groups in the two-hit assay. The sum of individual T-cell proportions specific for any of the three antigens was set to one and relative contribution of individual antigens is calculated. None of the individuals had a dominant (more than 50 % of IFN-γ positive T cells) Rv2628 specific T-cell response (open triangle region). HCs predominantly had either a Rv1733 dominant response (blue triangle region) or no dominance (center) (Figure 4, upper graph). Interestingly, tuberculosis patients showed two main phenotypes (Figure 4, lower graph). The majority had an E6/C10 dominant T-cell response (red triangle) but a considerable subgroup of QFT negative tuberculosis patients, 5 of 14 (36 %), had a Rv1733 dominant T-cell response (blue triangle). We concluded that *Mtb* latency antigens and especially Rv1733 may improve detection of *Mtb* infection in HCs and tuberculosis patients without detectable E6/C10 specific T cells.

## Discussion

In this pilot study we demonstrate the capacity of a two-hit long-term *in vitro* assay to improve detection of *Mtb* infection of tuberculosis patients and HCs from Ghana. QFT results were negative or indeterminate for the majority of tuberculosis patients and healthy contacts. This outcome confirmed own previous studies that showed low sensitivity of QFT tests for detection of *Mtb* infection in children with tuberculosis from Ghana (6). In contrast, a parallel study in children with tuberculosis and LTBI from Germany showed almost optimal sensitivity of the QFT (7). This suggested an effect occurring in a tuberculosis high-endemic country like Ghana and raised the question if low sensitivity was specific for children and/or acute tuberculosis. Here, we show that low QFT sensitivity is also found in adults from Ghana and that both, tuberculosis patients and HCs, were affected. For tuberculosis patients, impaired T-cell responses to PHA stimulation was seen as an additional effect that increased the frequency of indeterminate test results in combination with high IFN-γ background. Generally impaired T-cell functions has been described and this may contribute to low QFT sensitivity for *Mtb* specific T-cells (25).

In the present study, we established a QFT comparable *in vitro* assay (QFT_*in-vitro*_) to characterize mechanisms involved suboptimal QFT sensitivity. It turned out that intracellular IFN-γ measurements [after including costimulatory antibodies (23)] and purification of PBMCs only marginally improved sensitivity for *Mtb* specific T cells for healthy contacts. However, intracellular IFN-γ measurements in purified PBMCs largely reversed impaired T-cell response to PHA in tuberculosis patients, indicating that serum factors at least partially accounted for impaired PHA induced T-cell response. A possible explanation would be high levels of inflammatory and regulatory cytokines found in serum of patients with acute tuberculosis (26, 27). Recently, we showed that high serum IL-10 and IL-6 levels were accompanied by constitutive STAT3 phosphorylation and SOCS3 expression (27). Especially SOCS3 correlated negatively with T-cell IFN-γ production and may therefore contribute to low PHA response (27).

Intracellular IFN-γ measurements also improved detection of *Mtb* PPD-specific T cells as compared to the QFT_*in-vitro*_ assay. This suggested that inclusion of additional antigens could improve the sensitivity and that short-term assay conditions may be suboptimal for detection of *Mtb* infection in Ghana. Several studies indicated higher sensitivity of long-term (i.e., five to seven days) *in vitro* stimulation using latency *Mtb* antigens (20, 21). These antigens have been well characterized as immunodominant in different *Mtb* exposed populations across the world (28–32).

Previously, we demonstrated that the two-hit assay detected T-cell responses against latency *Mtb* antigens in LTBIs as well as in a minor subgroup of tuberculosis patients but not in IGRA-negative controls (19). We did not include controls without a known recent history of *M. tuberculosis* contact in the present study since identification of non-*M. tuberculosis* infected controls is difficult in a country with high tuberculosis prevalence like Ghana. In addition, we demonstrated that the QFT test fails to detect T-cell responses in confirmed *Mtb* infected patients and, hence, QFT negative results do not reliably exclude *Mtb* infection in Ghana. Like for most Sub-Saharan countries, children are BCG vaccinated at birth and this limits the significance of the TST. Against this background, large cohorts are needed to evaluate differences between potential LTBI (IGRA-positive or negative) and IGRA-negative donors (with an unknown history of *Mtb* infection) with sufficient statistical power. This was not possible as part of this pilot study but will be performed as part of a follow-up study. We concluded from the present study that QFT tests do not reliably exclude or confirm *Mtb* infection in Ghana and that more sensitive assays are needed to diagnose *Mtb* infection. The two-hit assay is a candidate for an immune test with higher sensitivity but needs to be evaluated by future studies.

In the present study, two-hit stimulation with the same respective antigen markedly enhanced T-cell response against E6/C10 in both study groups. Notably, tuberculosis and HCs without T-cell response in short-term assays benefited most from the re-stimulation with E6/C10. Previous studies predominantly focused on IGRA positive tuberculosis patients and healthy control cohorts to determine *Mtb* latency antigen specific T-cell responses (9). QFT/IGRA negative (or indeterminate) individuals were excluded in the majority of these studies, to avoid inclusion of misdiagnosed tuberculosis patients and non-*Mtb* infected HCs. But by implication, these studies excluded tuberculosis patients and HCs that were false negative in QFT/IGRA. Since previous studies clearly demonstrated that also IFN-γ negative CD4^+^ T cells producing alternative cytokines contribute to immunity against *Mtb* (6, 30), we hypothesize that negative QFT/IGRA results do not preclude Mtb infection.

Immune-based assays with high sensitivity are of paramount importance to allow early interventions strategies especially in *Mtb* infected HCs with high risk to develop active tuberculosis (e.g., young children, immune compromised patients). For young children, BCG effects on *Mtb* antigen specific immune responses are generally possible but previous studies did not see effects of BCG vaccination on T-cell responses against latency antigens (33, 34).

To our knowledge this is the first study to show that long-term *in vitro* culture with two-hit stimulation may improve detection of *Mtb* infection in QFT negative (or indeterminate) tuberculosis patients and HCs. The current study design did not allow to directly prove of *Mtb* infection for HCs. However, we used strict inclusion criteria for HCs to ensure tight and long-term contact to a known index patient, this way strongly increasing the likelyhood of being *Mtb* infected. Therefore, we assume that at least the majority of HCs are LTBI as indicated by the two-hit assay. Future studies -including follow-up of HCs and identification of tuberculosis progressors- will address the question if differences in the response against E6/C10 in two hit assays may contribute to diagnosis of *Mtb* infection in HCs.

For HCs, future studies will determine if high sensitivity of two-hit responses (100% in our cohort) is accompanied by high specificity to detect LTBI within HCs. These results may contribute to the decision about early prevention therapy in highly tuberculosis susceptible individuals.

Several studies have addressed the question if *Mtb* latency antigens can be used as T-cell targets of immune assays in *Mtb* infection [reviewed in (9)].We selected the most promising candidates from those and own previous studies (19). Rv2628 and Rv1733 were identified by others (12, 14–16) and also the capacity to discriminate tuberculosis patients from LTBI has been described for Rv2628 (15, 18). Our results confirm these studies and render Rv2628 and Rv1733 proteins most promising candidates for *Mtb* immune assays. T-cell responses were less frequently found for Rv2031 and Rv3407, although differences between the study groups for Rv3407 were detected. In general, the overlap of promising latency antigen candidates found in previous studies was moderate (9). This may be due to differences in the genetic background of the study populations examined and different assay types used. Application of a small group of latency antigens covering this heterogeneity and optimization of assay conditions for individual antigens could circumvent this problem. In addition, a group of antigen candidates can include antigens which are more generally recognized (e.g., Rv1733, E6/C10) as well as those with a higher capacity to discriminate (e.g., Rv2628).

Future studies will need to address the question whether immune response against latency antigens, Rv2628 and Rv1733, can help to predict tuberculosis disease progression in recently *Mtb* infected HCs. This way immune correlates of tuberculosis risk vs. reduced risk can be identified with implications for treatment intervention and vaccine design strategies. This long-term stimulation and the requirement of sophisticated flow cytometry measurement restricts the applicability of this assay for clinical routine. Therefore, this test may only be performed in well-equipped research laboratories accessible for few hospitals in Africa like in Kumasi/Ghana. Especially, potentially *Mtb* infected individuals with high risk of tuberculosis disease progression (i.e., young children, HIV co-infected individuals, patients treated with anti-TNFα immune modulatory drugs) may benefit from this test.

## Data Availability

All datasets generated for this study are included in the manuscript and/or the Supplementary Files.

## Ethics Statement

The present study received approval from the Committee on Human Research, Publication and Ethics (CHRPE/AP/023/18; CHRPE/221/14) at the School of Medical Sciences (SMS) at the Kwame Nkrumah University of Science and Technology (KNUST) in Kumasi, Ghana. All study subjects gave written informed consent prior to recruitment and for children written informed consent was provided by their parents or legal guardians.

## Author Contributions

EA, CL, and AG performed the experiments and contributed to analyses. EM, EO-D, RP, NN, and MJ designed the study. EA, EO-D, and RP recruited patients and contacts. KF and TO provided antigens and expertise. NN, RP, and MJ supervised the study. EA and MJ analyzed the data and wrote the manuscript.

### Conflict of Interest Statement

The authors declare that the research was conducted in the absence of any commercial or financial relationships that could be construed as a potential conflict of interest.
