# Supplementary material for: Two-Hit in vitro T-Cell Stimulation Detects Mycobacterium tuberculosis Infection in QuantiFERON Negative Tuberculosis Patients and Healthy Contacts From Ghana
Source: Front Immunol. 2019 Jul 3;10:1518. doi: 10.3389/fimmu.2019.01518 (PMC6616195; doi:10.3389/fimmu.2019.01518)
Supplement: Supplementary file 1 [file Data_Sheet_1.PDF]

Supplementary Figure 1

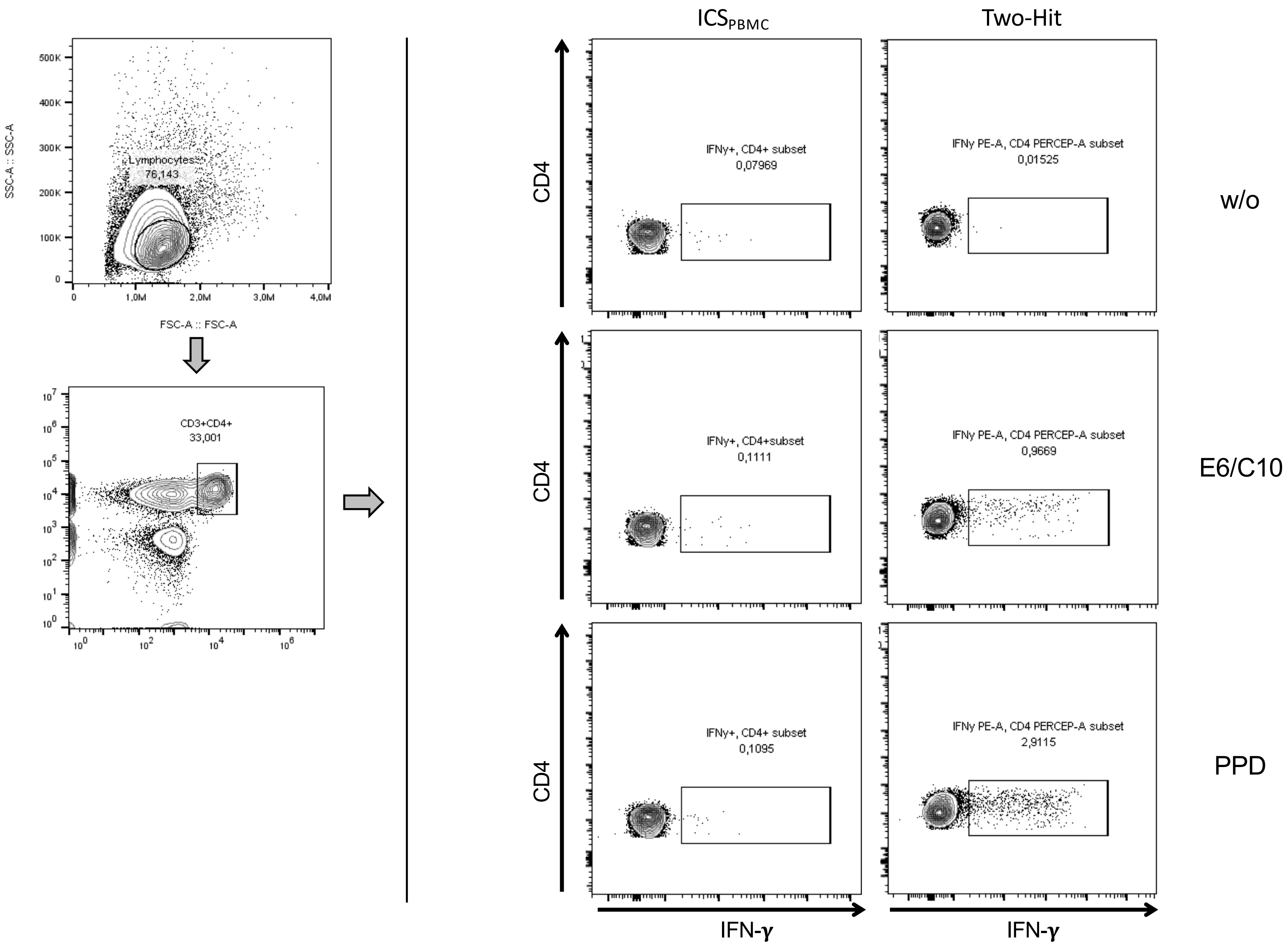

A representative data set depicting analysis procedure of flow cytometry data for intracellular cytokine measurement of ICS$_{PBMC}$ and the two-hit assay for an individual donor is shown.

Supplementary Figure 2

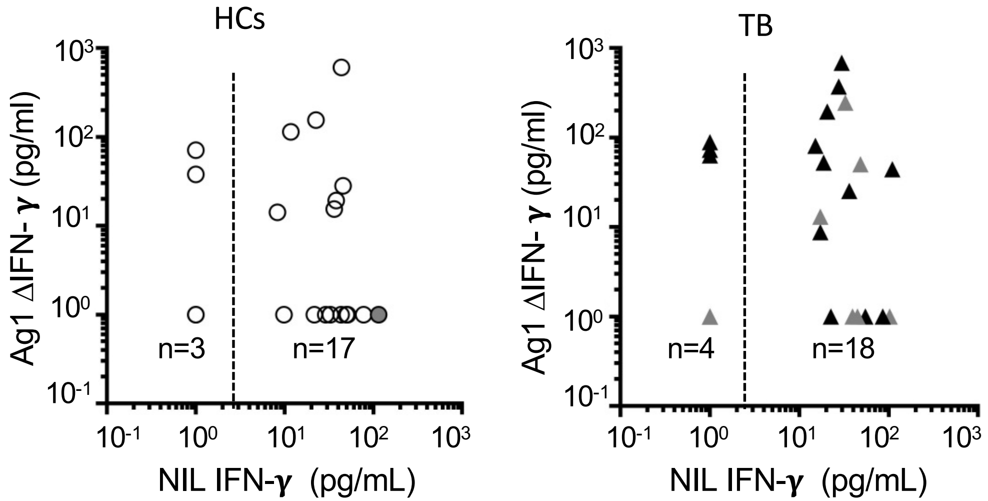

NIL (x-axis) and Ag1 (y-axis) induced IFN-γ concentrations of QFT supernatants are shown for tuberculosis patients (TB) patients and healthy contacts (HCs). Donors with indeterminate QFT are indicated by grey color. Numbers (n) of donors with low and high NIL IFN-γ values are given for both study groups

Supplementary Figure 3

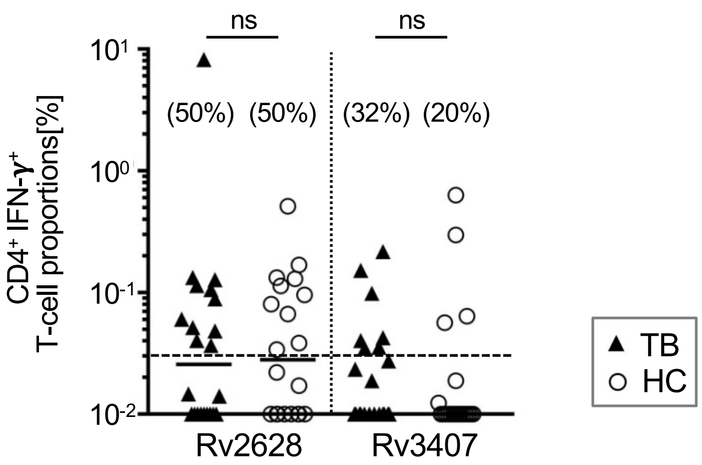

IFN-γ positive CD4⁺ T-cell proportions specific for latency *Mtb* antigens Rv2628 and Rv3407 after short-term *in vitro* stimulation. Symbols indicate mean values of duplicates for individual tuberculosis patients (black triangles) and HCs (open circles). Proportions of responders (according to the threshold of 0.03%) are indicated in brackets. Mann-Whitney *U*-test (two-tailed) indicated no significant differences between the study groups. ns: not significant.

Supplementary Figure 4

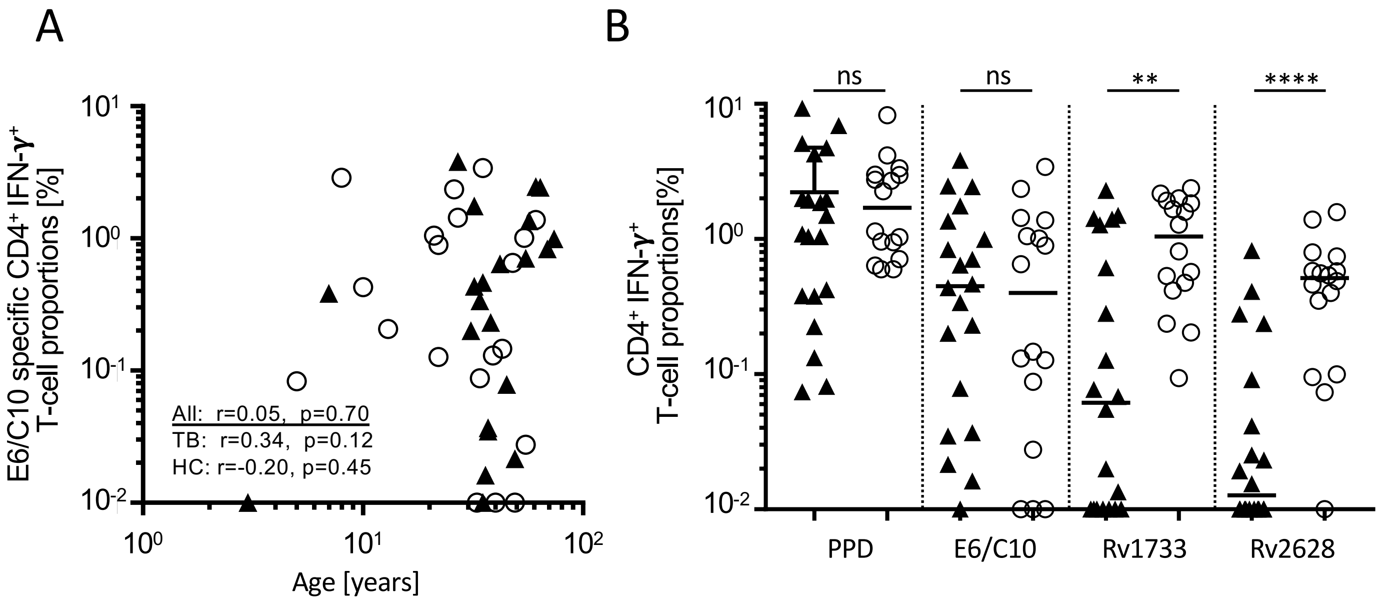

IFN-γ positive CD4+ T-cell proportions specific for *Mtb* antigens after two-hit stimulation. Symbols indicate mean values of duplicates for individual tuberculosis patients (black triangles) and HCs (open circles). (A) E6/C10 specific T-cell proportions (y-axis) and donor age (x-axis) are depicted and association was determined using the Spearman Rank test for all donors and both study groups separately. Correlation coefficients (r) and nominal p-values are given. (B) CD4+ T-cell proportions after two-Hit stimulation with PPD, E6/C10, Rv2628, and Rv3407 are depicted for study subgroups of adult (>18y) donors. Nominal p-values for the Mann-Whitney *U*-test (two-tailed) were calculated and shown as ** for p<0.01 and **** for p<0.0001.

Supplementary Table 1: Evaluation criteria for QFT.

Interpretation	ΔTB1 specific antigen response[1]<br>(pg/ml)[2]	Nil control<br>(pg/ml)[2]	ΔMitogen control[1]<br>(pg/ml)[2]
**Positive**	≥ 17.5<br>(and ≥ 25% of Nil)	≤ 400	any
**Negative**	< 17.5<br>OR<br>≥ 17.5 and < 25% of Nil	≤ 400	≥ 25
**Indeterminate**	< 17.5<br>OR<br>≥ 17.5 and < 25% of Nil	≤ 400	< 25
	any	> 400	any

[1]Corrected for Nil response. [2]IFN-γ concentration converted by 1 IU/ml = 50 pg/ml. The table was adopted from manufacturers' instructions.

Supplementary Table 2: Evaluation criteria for $QFT_{in\text{-}vitro}$.

Interpretation	ΔE6C10 specific response[1] (pg/ml)	ΔMitogen control[1] (pg/ml)
**Positive**	≥ 1.5 (and ≥ 25% of Unstim.)	any
**Negative**	< 1.5 OR ≥ 1.5 and < 25% of Nil	≥ 5
**Indeterminate**	< 1.5 OR ≥ 1.5 and < 25% of Nil	< 5
	any	any

[1]Corrected for unstimulated response
